# Microbial communities of the upper respiratory tract in mild and severe COVID-19 patients: a possible link with the disease course

**DOI:** 10.3389/frmbi.2023.1067019

**Published:** 2023-05-29

**Authors:** Julia S. Galeeva, Elizaveta V. Starikova, Dmitry E. Fedorov, Alexander I. Manolov, Alexander V. Pavlenko, Dmitry N. Konanov, Danil V. Krivonos, Vladislav V. Babenko, Ksenia M. Klimina, Vladimir A. Veselovsky, Maxim D. Morozov, Ilshat R. Gafurov, Raushaniya F. Gaifullina, Vadim M. Govorun, Elena N. Ilina

**Affiliations:** ^1^ Research Institute for Systems Biology and Medicine, Department of Mathematical Biology and Bioinformatics, Moscow, Russia; ^2^ Lopukhin Federal Research and Clinical Center of Physical-Chemical Medicine of Federal Medical Biological Agency, Department of Molecular Genetics of Microorganisms, Moscow, Russia; ^3^ Kazan Federal University, Department of Molecular Medicine and Biology, Kazan, Russia

**Keywords:** COVID-19, 16S, human microbiome, oropharyngeal swabs, SARS-CoV-2, upper respiratory tract

## Abstract

The microbiota of the respiratory tract remains a relatively poorly studied subject. At the same time, it is involved in modulating the immune response to infectious agents in the host organism, just like the intestinal microbiota. A relationship between the composition of the respiratory microbiota and the likelihood of development and the severity of COVID-19 may be assumed. In this study, we applied the 16S rRNA metagenomic sequencing to analyze the oropharyngeal swabs from 120 COVID-19 patients collected during the first and the second waves of the COVID-19 epidemic in Russia. Differential abundance analysis with respect to comorbidities suggested association of *Neisseria oralis, Neisseria mucosa*, unidentified *Veillonella* spp.*, Lautropia mirabilis* species with more severe lung damage, and *Streptococcus salivarius*, *Capnocytophaga sputigena* and *Haemophilus parahaemolyticus* with a milder course of the disease. We hypothesize that the latter bacteria (or some of them) might be beneficial for the respiratory tract and might be able to alleviate the course of the COVID-19 disease.

## 1 Introduction

Commensal microorganisms of the respiratory tract affect the body’s ability to withstand a threat of viral infections (Abt et al., 2012; Lee et al., 2019). This might be due to the modulatory effect of microbes on the immune system (Abt et al., 2012) or direct impact of the microbial metabolites on the viral life cycle (Kamio et al., 2015).

At the same time, certain bacteria can promote viral infections (Bellinghausen et al., 2016), and secondary bacterial infections can lead to serious complications and mortalities (Morens et al., 2008; Lansbury et al., 2020). Viral infections in turn can influence the bacterial community, sometimes promoting bacterial biofilm formation and secondary infections (Hendricks et al., 2016; Hendricks et al., 2021). The relationship between respiratory microbiota and viral infections remains an important research subject (Man et al., 2017; Hendricks et al., 2021).

Until recently, the microbiota of the upper respiratory tract was poorly studied, especially in adults (Dubourg et al., 2019). This situation is now changing due to the current COVID-19 pandemic. Previous study has shown the dynamical nature of microbial community types of the upper respiratory tract during COVID-19 infection (Xu et al., 2021). A number of studies described differences between patients with COVID-19 and healthy people or differences between patients with different levels of disease severity (Rosas-Salazar et al., 2021; Soltani et al., 2021) among others, (see (Yamamoto et al., 2021) for a short review). The results of various studies are not always consistent. It might be due to the phenotypic differences between subjects (e.g. comorbidities), as well as seasonal and geographical factors and different techniques used in data sampling and analysis.

When studying the current COVID-19 samples, researchers use different approaches. In a study performed in Nashville, Tennesy (Rosas-Salazar et al., 2021), the researchers examined 38 COVID-19 patients and 21 uninfected controls by 16S rRNA metagenomics. They obtained amplicon sequence variants (ASVs) using DADA2 method and applied DESeq2 to infer ASVs associated with the infection and also with high viral load. They have noted that ASVs associated with COVID-19 disease and high viral load belonged to *Peptoniphilus lacrimalis*, *Campylobacter hominis*, *Prevotella copri*, and an *Anaerococcus* unclassified amplicon sequence variant. At the same time, *Corynebacterium* unclassified, *Staphylococcus haemolyticus*, *Prevotella disiens*, and two *Corynebacterium_1* unclassified amplicon sequence variants were associated with healthy status and low viral load during COVID-19.

In another study (Ren et al., 2021), the researchers performed metatranscriptome sequencing of 588 oropharyngeal swab samples collected from 192 COVID-19 patients. The authors found that *Streptococcus* genus was enriched in recovered patients, whereas potential pathogens, including *Candida* and *Enterococcus*, were more abundant in patients that have died of infection. They also noted that high abundance of *Streptococcus* and particularly of *S. parasanguinis* at the moment of patient’s admission was a strong predictor of fatality. Researchers in India (Devi et al., 2022) analyzed 198 nasopharyngeal and/or throat swabs from COVID-19 patients using WGS sequencing. The patients were categorized into four groups based on disease severity and outcome: mild, moderate, severe and lethal. The researchers discovered significant transcript abundance of *Achromobacter xylosoxidans* and *Bacillus cereus* in the lethal group, *Leptotrichia buccalis* in the severe, *Veillonella parvula* in the moderate, and *Actinomyces meyeri* and *Halomonas* sp. in the mild COVID-19 patients.

Respiratory microbiome has geographic and climatic characteristics, so it is important to study its relationship with COVID-19 disease in different regions. In order to avoid spurious correlations, other diseases and patients’ habits should be also taken into account when analyzing associations between microbiome composition and the course of COVID-19 infection.

In our work, we describe the composition of the respiratory microbiome in 120 inpatients with COVID-19. We performed the 16S rRNA metagenomic analysis of oropharyngeal swabs and reconstructed metabolic pathways using the PICRUST2 tool to find associations with the disease severity.

We aimed to identify microorganisms and KEGG pathways associated with the course of the disease using differential abundance testing with patients’ metadata as covariates. We hypothesize that some of the identified microorganisms might have a protective role in the respiratory tract given the wide geographical and age ranges of COVID-19 patients involved in this study along with the comorbidities considered, we believe that the obtained results can be reproduced in further research.

## 2 Materials and methods

### 2.1 Samples and data collection

The oropharyngeal swabs of COVID-19 patients were collected between the May of 2020 and March of 2021 in four cities of the Russian Federation (Moscow, Nizhny Novgorod, Kazan and Irkutsk) featuring the following medical centers: Kazan Federal University, Federal State Budgetary Educational Institution of Higher Education «Privolzhsky Research Medical University» of the Ministry of Health of the Russian Federation, Federal State Public Scientific Institution «Scientific Сentre for Family Health and Human Reproduction Problems», Federal State Budgetary Institution of Healthcare Hospital of the Russian Academy of Sciences (Troitsk), Burnasyan Federal Medical Biophysical Center of Federal Medical Biological Agency, City Clinical Hospital named after S.I. Spasokukotsky of Moscow Healthcare Department, Federal Research and Clinical Center of Specialized Medical Care and Medical Technologies, Federal Biomedical Agency of the Russian Federation (Galeeva et al., 2022).

The study included both inpatients and outpatients diagnosed with COVID-19 who had a confirmed PCR test positive for SARS-CoV-2. All the patients have signed informed consent to participate in the study. The study did not include patients diagnosed with cancer.

For each patient we gathered the following information: computer tomography severity score (CT index), percent of lung tissue affected, body temperature, oxygen saturation level, respiratory rate, heart rate, systolic and diastolic blood pressure, consciousness and the need for additional oxygen supply. The following metadata were collected based on questionnaires: whether or not the patient has health disorders such as obesity, diabetes, chronic obstructive pulmonary disease (COPD), inflammatory bowel disease (IBD), arthritis, tuberculosis, hypertension, coronary artery disease (CAD), chronic heart failure, asthma.

Oropharyngeal swabs were collected from all participants in an ambulatory or hospital setting using a dry rayon swab. After collection, the samples were stored at -70 degrees Celsius. Оutpatients’ swabs were collected on the day of the first visit to the doctor. Inpatients’ swabs were collected on the day of the admission (the first time point), and on the day of release (the second time point).

### 2.2 16S rRNA sequencing

Nucleic acids were extracted using the MagMAX DNA Multi-Sample Ultra 2.0 Kit and KingFisher™ Purification System (Thermo Fisher Scientific, USA) according to the manufacturer’s protocol. The DNA was subsequently quantified on Qubit 4 fluorometer by Quant-iT dsDNA BR Assay Kit (Thermo Fisher Scientific, USA).

The library preparation was done according to 16S Metagenomic Sequencing Library Preparation Illumina protocol. Briefly, the extracted DNA was amplified using the 341F and 801R primers, which are complementary to the V3-V4 region of 16S rRNA gene and contain 5’-illumina adapter sequences. During the next step, individual amplicons were PCR–indexed and pooled. DNA libraries were sequenced on a MiSeq instrument (Illumina, USA) using the Miseq reagent kit v3 (Illumina, USA).

### 2.3 16s rRNA data processing

Leftover adapters were removed using Trimmomatic v0.36 (Bolger et al., 2014; Devi et al., 2022), and quality filtering of reads was performed with *filterAndTrim* function from DADA2 package (Callahan et al., 2016). Denoising, merging and chimera removal was carried out with DADA2 v1.24.0 software with following parameters: learnErrors: nbases=1e+09, randomize=TRUE, MAX_CONSIST=2, dada: pool = TRUE, mergePairs: minOverlap=18, removeBimeraDenovo: allowOneOff=FALSE, method=“consensus”.

Taxonomy annotation was carried out against the SILVA v138 reference database (Pruesse et al., 2007).

Potential contaminants were removed with the “frequency” method using the package *decontam* (Davis et al., 2018) version 1.10.0. In total the 1600 samples were decontaminated (504 samples from this project and 1086 samples from other projects with similar objects for analysis) to better identify contaminant sequences. Following decontamination, we introduced the variable “contamination” to indicate the proportion of sequences identified as contaminants and subsequently removed from the analysis, with the following levels: low (0-5%), middle (5-20%), high (20% and higher).

The resulting dataset contained 6235 ASVs, with 4482 ASVs for the target dataset. The mean number of reads per sample was 16101. Samples with <1,000 reads were removed.

PICRUSt2 v2.5.1 tool was used to predict functional abundances from 16S data and a reference genome database with stratified output. PICRUSt2 identified 7491 metabolic pathways which were filtered down to the 3663 most abundant pathways for downstream analysis.

The general scheme of sample processing is shown on **Supplementary Figure 1**.

### 2.4 Statistical analysis

Statistical analysis of the microbiome was performed in R (v.4.0.5) using vegan (Dixon, 2003) and phyloseq (McMurdie et al., 2013) packages.

We applied *core_members* function from the microbiome package with the following criteria (detection=5 prevalence=5/100) to filter out the low-represented ASVs and obtained 290 ASVs.

For further statistical analysis 290 ASVs were agglomerated into 146 ASVs on the phylogenetic tree using *tip_glom* function with h=0.05 (cophenetic distance) from the phyloseq package.

The diversity composition of the bacterial microbiome was evaluated using α-diversity (Shannon index) using *plot_richness* function from the phyloseq package.

Associations of taxa with host parameters were identified by using permutational multivariate analysis of variance (PERMANOVA). PERMANOVA with 1000 permutation tests was run on UniFrac distance by using *adonis* function from the vegan package.

Differential abundance analysis at the ASV level was performed to identify ASVs differentially abundant in mild and severe groups of patients using the DESeq2 package (Varet et al., 2016) in R and Songbird utility (Varet et al., 2016; Morton et al., 2019) in Python. When using the DESeq2 tool, statistical significance of log2FoldChange was assessed using the default Wald test with Benjamin-Hochberg p-value correction. Cut-off for all significant tests was set at p.adjusted < 0.05 and Log2FoldChange >= 1.5. Songbird was also used to determine differential rankings of microbes between mild and severe groups. The intersection in the differentially represented ASVs between these two instruments was chosen for further analysis.

To estimate the statistical significance of the distribution of differentially abundant ASVs between first and second time points in the groups of severe and mild patients, the Wilcoxon rank sum test was applied.

Differentially abundant pathways in mild and severe groups of patients were determined by the Songbird package.

## 3 Results

### 3.1 Study cohort

**Figure 1:**
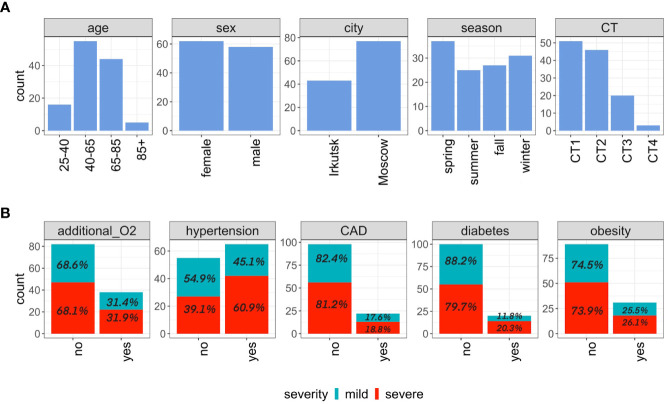
Study cohort overview. **(A)** Distribution of patients by age, sex, city of sampling, season of sampling and CT score for hospital patients included in the study. **(B)** The clinical characteristics of inpatients are shown: the need for additional oxygen supply as well as the presence of hypertension, coronary artery disease (CAD), diabetes, obesity as a concomitant disease.

In final, the 174 samples from 120 COVID-19 inpatients were involved (**Supplementary Figure 1**). For 54 patients both samples collected on admission to the hospital and on discharge from the hospital were available. The included samples were collected during the decay of the first wave and the entire period of the second wave of COVID-19 incidence in Moscow and Irkutsk (**Figure 1A**).

These 120 patients were between 18 and 85 years old, 62 females and 58 males. Among them the 51 (51/120, 42.5%) possessed CT1 severity score on admission to the hospital, the 46 (46/120, 38.3%) - CT2, the 20 (20/120, 16.7%) - CT3 and the three (3/120, 2.5%) - CT4 (**Figure 1A**). CT-based severity classification system reflects the extent of COVID-associated lung abnormalities seen on the CT scans, from up to 25% (CT1) to 50% (CT2), 75% (CT3) and up to 100% (CT4) (Morozov et al., 2020).

For further comparison analysis all 120 patients were divided into two groups - patients with mild COVID (51 subjects with CT1 severity score) and patients with moderate/severe COVID (69 patients with CT2, CT3 and CT4 severity score). The last one was named “severe” group.

We analyzed the distribution of some comorbidities in the groups of patients with mild and severe COVID-19. **Figure 1B** shows that compared with the mild group, patients with severe disease are more likely to be diagnosed with hypertension (60.9% versus 45.1%) as well as diabetes (20.3% versus 11.8%).

### 3.2 Taxonomic composition of oropharyngeal microbiome of SARS-CoV-2 infected patients

146 ASVs corresponding to 36 families with a mean of 16101 reads per sample. The microbial composition of samples from 120 patients with mild to severe COVID-19 is composed of such dominant family as *Campylobacteraceae*, *Lactobacillaceae*, *Gemellaceae*, *Neisseriaceae*, *Veillonellaceae*, *Streptococcaceae* and *Prevotellaceae* (**Supplementary Figure 2**).The taxonomic profile at ASV level is shown in **Supplementary Figure 3**.

### 3.3 No significant differences in alpha-diversity between mild and severe groups of patients infected Covid-19

**Figure 2:**
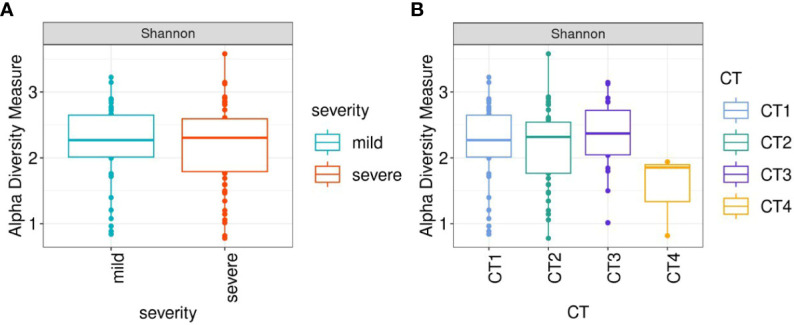
Box-plots illustrate alpha diversity by Sannon index in bacterial microbiomes of 120 inpatients. **(A)** samples from mild (n=51), severe (n=69) groups **(B)** samples from patients with different CT scores (CT1 = 59; CT2 = 46; CT3 = 20; CT4 = 3). Median values and interquartile ranges have been indicated in the plots.

To evaluate alterations in the microbiota community structure between each group, the microbial alpha diversity was measured by Shannon metric as shown in **Figure 2**. The results show that there are no differences in alpha diversity indices between mild and severe groups of patients (**Figure 2A**).

When comparing patient groups based on CT scans of lung abnormalities, patients with a CT4 score showed decreased alpha diversity compared to patients with CT1, CT2, and CT3 (**Figure 2B**).

### 3.4 Testing the association between the oropharyngeal microbiome of patients infected SARS-CoV-2 and covariates

**Figure 3:**
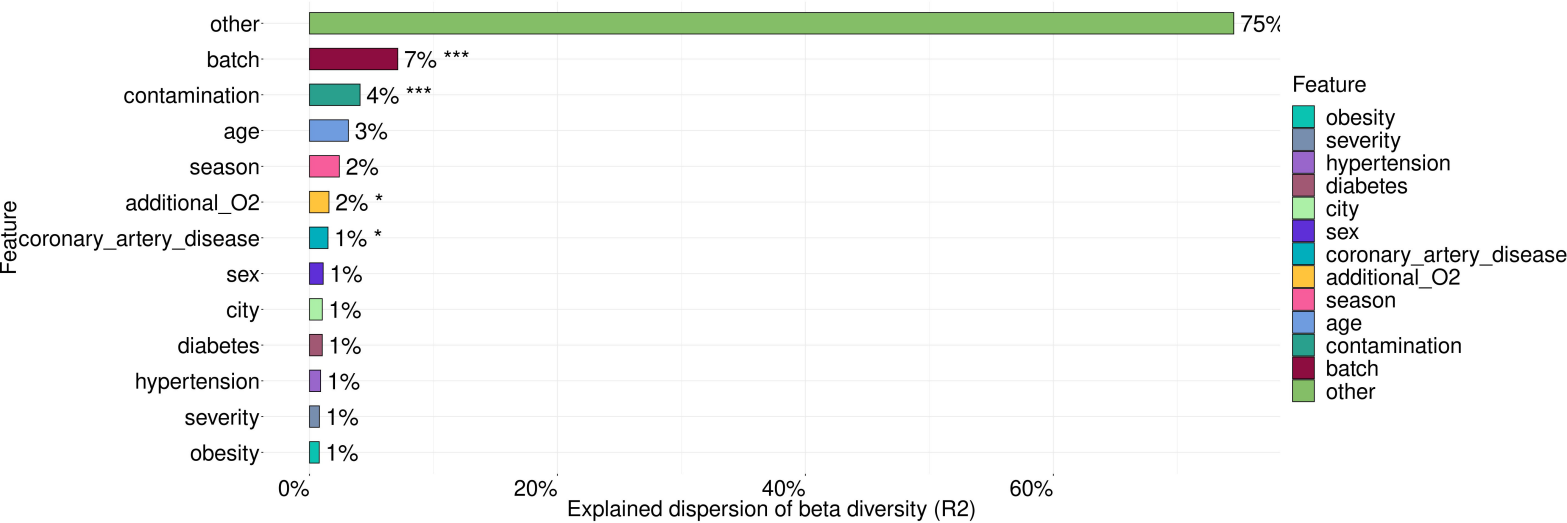
The variance in microbiome composition explained by metadata factors. Asterisks indicate the level of significance with the following thresholds: (*) = 0.05, (***) = 0.001.

For testing the association between the microbiome and such covariates as batch, contamination, age, city, severity of lung damage based on CT scan, season of sample collection, the need of additional O2, hypertension, CAD, diabetes and obesity, we applied PERMANOVA analysis (**Figure 3**).

These covariates explained 26% of microbiome taxonomic composition with the largest contribution coming from batch (7%), contamination (4%), age (3%), season of sample collection (2%) and the need of additional O2. Statistically significant contributions were made by such covariates as batch, contamination, the need of additional O2 and coronary artery disease (CAD).

### 3.5 Microbial associations of disease severity with respect to age, sex, city, season of the sampling, sequencing batch and comorbidities

**Figure 4:**
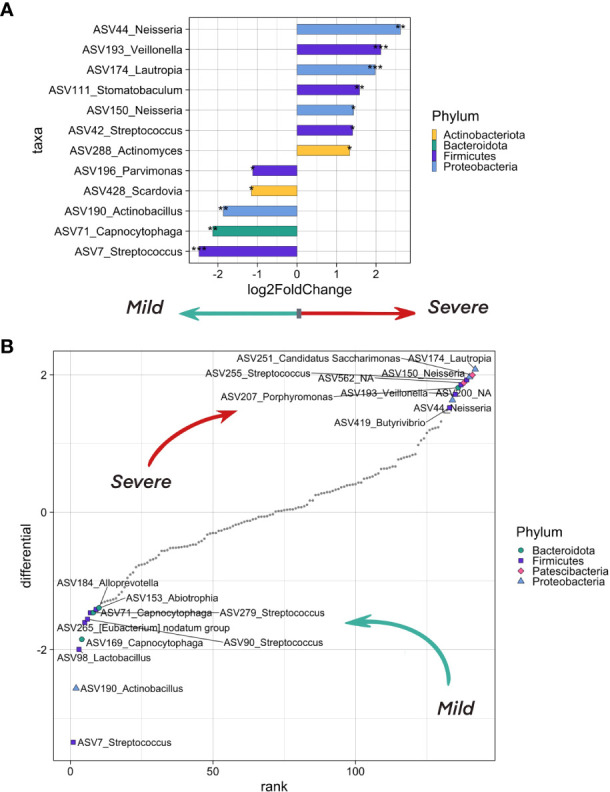
The differentially abundant taxa testing associations. **(A)** Bacteria on the ASV level that differ in abundance in inpatients with severe (CT >= 2) and mild (CT = 1) levels of lung damage. The analysis was carried out using the DeSeq2 method. Bacteria which are more abundant in more severe patients are shown in bars pointing to the right, in lighter patients - to the left. Asterisks indicate the level of significance with the following thresholds: (*) = 0.1, (**) = 0.05, (***) = 0.01. **(B)** Top ASV that are associated with COVID-19 severity as analyzed by the songbird utility. ASVs associated with a milder course of covid have lower differential values, with a more severe one - larger ones. The 10 most associated (in one direction and the other) ASVs have been selected, their phylum is marked in color and shape.

In order to search for the potential effect of oropharyngeal microbiome composition on the severity of COVID-19, we took into account the following metadata: age, sex and comorbidities of the patient, city and season of the sampling, and sequencing batch. We analyzed the differentially abundant genera between patients with mild (n=51) and more severe (n=69) levels of lung damage using the DESeq2 (**Figure 4A**) and Songbird methods (**Figure 4B**). Further, we selected ASVs associated with mild and severe disease courses of COVID-19 that overlap between outputs of these two tools.

We observed an overrepresentation of ASV7, ASV71 and ASV190 in samples from patients with mild lung damage (CT1). These ASVs belong to *Streptococcus*, *Capnocytophaga*, and *Actinobacillus* genera, respectively. When aligning ASV sequences on nt database using BLASTN, we have identified ASV7 as *Streptococcus salivarius*, ASV71 as *Capnocytophaga sputigena*, and ASV190_*Actinobacillus* as *Haemophilus parahaemolyticus* with 100% identity (**Supplementary Table 1**).

We found four ASVs from *Neisseria*, *Veillonella*, and *Lautropia* genera that were associated with severe lung damage (CT2-4), namely, ASV44 corresponding to *Neisseria mucosa*, ASV193 corresponding to uncultured bacterium from *Veillonella* genus, ASV150 corresponding to both *Neisseria oralis* (identity = 99.77%) and *Neisseria sp* (identity = 100%), and ASV174 corresponding to *Lautropia mirabilis* (identity = 99.6%) and uncultured bacterium from *Lautropia* genus (identity =100%) (**Supplementary Table 1**).

**Figure 5:**
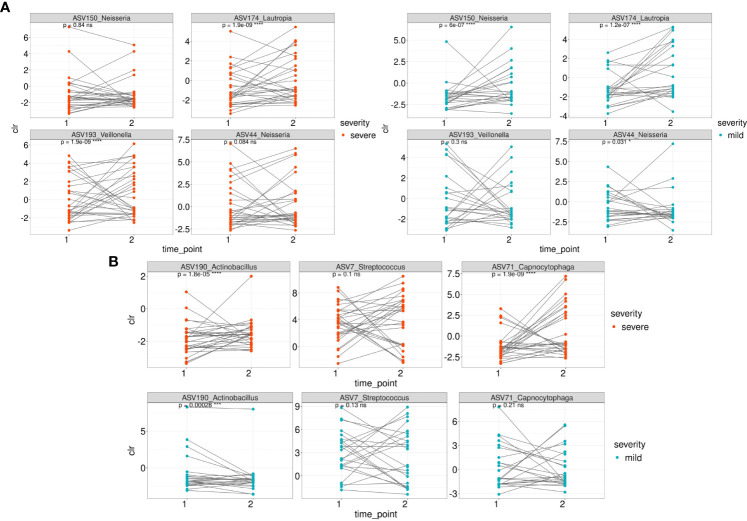
The dynamics of the relative abundance (CLR normalized) of ASVs that were associated with both mild and severe groups between 1 and second time point. **(A)** The dynamics of ASVs that were differentially over-represented in the group of severe patients between 1 and 2 time point in severe and mild groups **(B)** The dynamics of ASVs that were differentially over-represented in the group of mild patients between 1 and 2 time point in severe and mild groups.

We then traced the dynamics of the relative abundance of ASVs that were associated with both mild and severe (**Figures 5A, B**) patient groups whose samples were collected at the first and second time points (n=54). Taxonomic composition of 54 patients is shown in **Supplementary Figure 4**).

Among the ASVs that were differentially over-represented in the group of severe patients, the relative abundance of ASV150 (*Neisseria* sp*/Neisseria oralis*) was statistically significantly overrepresented in the mild patients at the second time point while the relative abundance of ASV44 (*Neisseria mucosa*) underrepresented (**Figure 5A**). ASV174 (*Lautropia genus/Lautropia mirabilis*) was statistically significantly overrepresented at the second time point of severe and mild patients. ASV193 (*Veillonella* genus) is overrepresented at the second time point of severe patients.

Regarding the ASVs associated with the mild course of the disease, the relative abundance of ASV71 (identified as *Capnocytophaga sputigena*) was significantly overrepresented at the second time point in patients from the severe group (**Figure 5B**). ASV190_Actinobacillus (identified as *Haemophilus parahaemolyticus*) was statistically significantly overrepresented at the second time point of mild and severe patients (**Figure 5B**).

### 3.6 Differentially abundant pathways in mild and severe groups of patients

**Figure 6:**
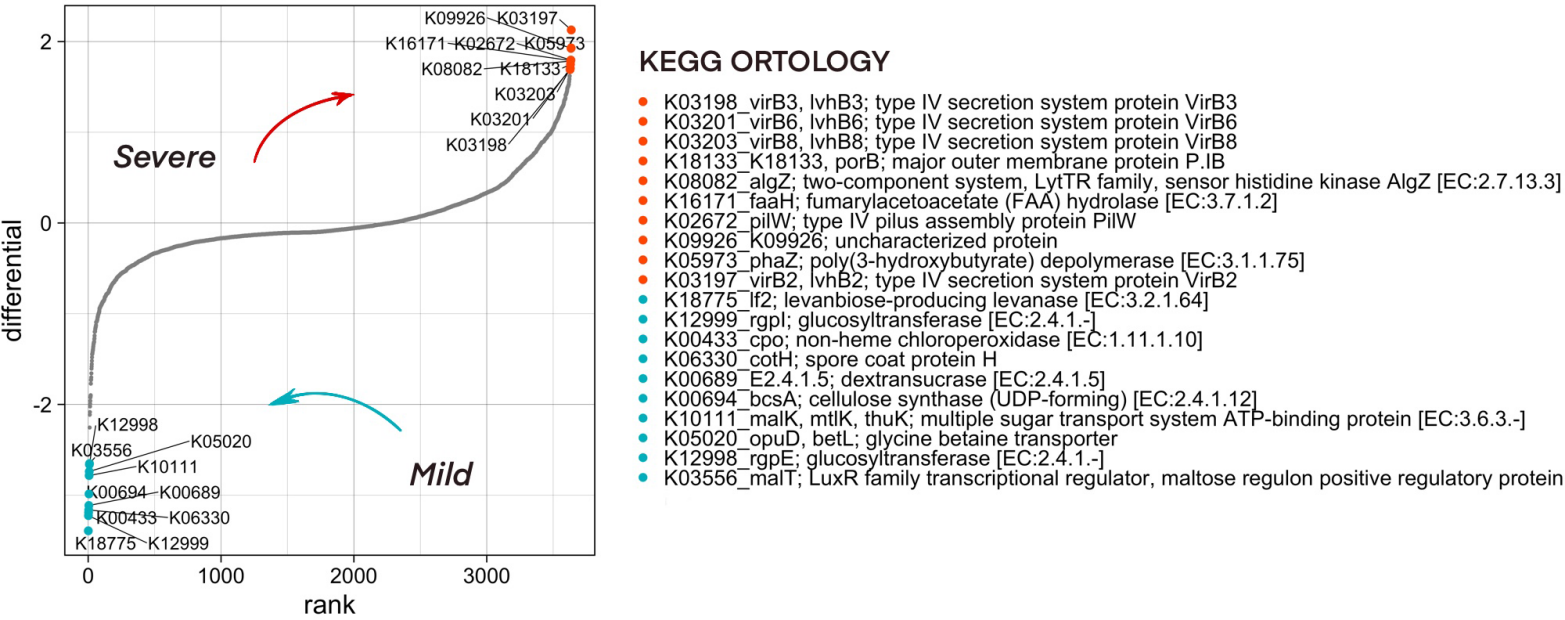
Predicted functionality that is associated with COVID-19 severity as analyzed by the songbird utility. Features associated with a milder course of COVID-19 have lower differential values, with a more severe one - larger ones. The 10 most associated (in one direction and the other) features have been selected, their association group is marked in color.

Functional diversity of bacterial community composition was revealed by PICRUSt2 assignment of KEGG orthology (KO) groups to KEGG pathways. In pharyngeal samples a total of 7491 different KO’s were identified. We then investigated metabolic pathways that were differentially abundant in the microbiome of mild and severe groups of COVID-19 patients using Songbird utility. We considered metadata such as batch, age, patient gender, city, sampling season, and comorbidities as covariates. The top pathways associated with microbiome of severe patients were dominated by secretory systems associated with DNA uptake and natural competence represented by K03197_VirB2, K03198_VirB3, K03201_VirB6, K03203_VirB8 and K18133_porB, K02672_pilW respectively (**Figure 6**).

To find the ASVs that contribute significantly to the metabolic pathways associated with microbiome of severe patients, a stratification analysis was performed using PICRUSt2. According to the results of the analysis of ASV33, ASV44, ASV82, ASV129, which belong to the *Neisseria*, and ASV130 from the *Lautropia* genera, contribute most to the representation of metabolic pathways associated with the severe course of the disease (**Supplementary Figure 5**).

## 4 Discussion

In our study, we applied the 16S rRNA metagenomic analysis to compare the taxonomic composition of the upper respiratory tract microbiota of COVID-19 patients at the time of admission to the hospital. Clinical samples from the current investigation were handled and sequenced with the similar ones from the other projects (**Supplementary Figure 1**).

Characterizing microbial communities from low-biomass samples like oropharyngeal swabs can be challenging due to the risk of contamination from exogenous DNA during sample collection and processing, which affects assay results (Salter et al., 2014). In our study, we used the decontam tool, but there are also a number of other recommendations and approaches for controlling contaminant sequences in low-biomass communities (Eisenhofer et al., 2019; Claassen-Weitz et al., 2020). We decontaminated the array of sequences from multiple projects simultaneously, enabling a more comprehensive cleaning of the array from contaminated sequences. We also excluded the identified contaminant sequences and introduced the variable “contamination” in the sample to account for this effect in the models. Our analysis focused solely on samples with complete metadata, including age, city, severity of lung damage based on CT scan, the season of sample collection, additional O2 requirements, hypertension, CAD, diabetes, and obesity. We employed these variables as covariates in our statistical analysis, although this approach did reduce our sample size, it ultimately allowed for a more robust statistical analysis. Finally, the 174 samples from 120 COVID-19 inpatients were left for analysis.

All the patients were divided into mild and severe groups according to their CT severity score (CT 1, 2, 3, or 4), which reflected the lung damage level at the time of hospitalization. This approach allowed us to consider the observed pneumonia as a community-acquired one and to avoid the finding of hospital-associated pathogens in the metagenomic samples.

We deliberately did not consider the option of comparing the oropharyngeal microbiome of healthy and diseased groups, as it was already performed in multiple studies (Xiong et al., 2021), (Iebba et al., 2021; Ren et al., 2021). It occurs that SARS-CoV-2 can also be found among healthy people that can be asymptomatic carriers of the virus. So, using asymptomatic (“healthy”) people as a control group can introduce biases into the analysis. Moreover, according to the principle of Anna Karenina, which has recently been frequently discussed in the context of metagenomic research, the microbiome of healthy people is less variable and more homogeneous than the microbiota of people with different diseases (Ma, 2020). Therefore, we decided to compare the upper respiratory tract microbiota between patients with different disease severity of SARS-CoV-2.

The oropharyngeal swabs were collected from COVID-19 patients during the first and the second waves of the epidemic, which were dominated by the “Wuhan’’ variant of SARS-CoV-2 (Awadasseid et al., 2021). Some papers discuss the effect of seasonality on the upper respiratory tract microbiota (Camarinha-Silva et al., 2012; Schoos et al., 2020; Zhao et al., 2022). In our study, the period of sample collection affected all seasons, which allows us to use seasonality as a covariate (**Figure 1**). The taxonomic composition of oropharyngeal swabs of COVID-19 patients is consistent with the results of studies about human oropharyngeal microbiota (Zaura et al., 2009; Bach et al., 2020). The taxonomic profile of our samples were found to be homogeneous, as confirmed by the heatmaps of **Supplementary Figure 3** and **Supplementary Figure 4**, where we observed the absence of clear clustering by such parameters as sex, age, city, seasonality, and batch effect.

Strikingly, the compared patient groups did not differ in the need for supplemental oxygen despite differences in the severity of lung damage. They also did not differ in the suffer of obesity, known to be a potential predictor of severe COVID-19 (Simonnet et al., 2020; Wang et al., 2022). Meanwhile, we observed the higher prevalence of hypertension and diabetes in the severe patient group. However, there is no conclusive evidence yet that hypertension is more common in patients with COVID-19 or that it might increase the risk of SARS-CoV-2 infection. Although hypertension is common among severe COVID-19 patients, it is more likely to be attributed to the vulnerability of middle-aged and older individuals to SARS-CoV-2 infection, according to this literature review (Shibata et al., 2020).

When comparing the alpha diversity of the upper respiratory tract microbiota of mild and severe patients, we observed no significant differences between these two groups. This is consistent with the results of (Hernández-Terán et al., 2021), but at the same time contradicts the results of the following study (Shilts et al., 2021), where the difference in the alpha diversity between patients with mild and severe disease was significant. The greatest difference was observed between CT4 and CT1-3 groups, where patients with СT4 had the lowest alpha diversity indices. Theoretically, this strong decrease in microbiota diversity could be related to inflammation in the airways and more frequent medication treatment of patients with severe pneumonia (Hong et al., 2021). Nevertheless, due to the restricted number of samples (n = 3) and the lack of information on drug treatment, we can neither confirm nor deny this assumption.

SARS-CoV-2 infection strongly affects the human immune system, causing a cytokine storm (Jose et al., 2020), activation of monocytes and macrophages, which in turn affects the human upper respiratory tract microbiome (Merad et al., 2020). COVID-19 can cause changes in the airway epithelium, increase local inflammation, and promote adhesion of respiratory pathogens. In our study, we identified bacterial drivers of patients with mild (CT1) and more severe (CT >= 2) lung damages, taking into account the metadata described above as covariates.

We found that ASVs identified as *Streptococcus salivarius*, *Capnocytophaga sputigena*, and *Haemophilus parahaemolyticus* were significantly more prevalent in patients with mild lung damage. These three bacteria belong to genera that are widely distributed in the human oropharyngeal microbiome.

*Haemophilus parahaemolyticus* is considered as a natural oropharyngeal commensal in humans, which can be pathogenic in certain cases. In a number of studies (Ren et al., 2021; Soffritti et al., 2021) that compared the microbiomes of healthy people and patients infected with SARS-CoV-2 virus, the genus *Haemophilus* and in particular *Haemophilus parahaemolyticus* species was associated with the healthy subjects.

*Capnocytophaga* is a commensal genus that is a part of the normal bacterial flora of the human oral microbiome. *Capnocytophaga* spp. and *Capnocytophaga sputigena* in particular often act as opportunistic pathogens associated with poor oral hygiene and periodontitis. At the same time, they occur in groups of healthy people and are closely associated with other bacteria (Idate et al., 2020; Jolivet-Gougeon et al., 2021; Ma et al., 2022). In the article (Soffritti et al., 2021) have reported an increased abundance of *Capnocytophaga sputigena* in SARS-CoV-2 patients as compared to healthy controls. In addition, *Capnocytophaga* spp. and other oral opportunists have been found in the BALF of the COVID-19 patients (Bassis et al., 2015; Bao et al., 2020; Wu et al., 2020).

*Streptococcus salivarius* is also a part of the commensal microflora of the upper respiratory tract. At the same time, some strains of this species are able to regulate the acid-alkaline balance of the oral cavity through the production of alkali. This property can have an antagonistic effect on a number of opportunistic species like *Streptococcus pyogenes*, *Streptococcus pneumoniae*, *Moraxella catarrhalis* and *Haemophilus influenzae* that prefer a more acidic environment and can cause a number of respiratory infections(Aebi, 2011; Zupancic et al., 2017; Abranches et al., 2018; Di Pierro, 2020; Wen et al., 2020).

Speaking about the patients with severe lung damage (CT2-4), we found *Neisseria, Veillonella* and *Lautropia* genera to be overrepresented in their oropharyngeal samples. All the three genera are widespread in the human oropharynx and belong to the commensal microflora (Zaura et al., 2009; Bach et al., 2020).

The *Neisseria* genus is one of the most common genera in the human oropharynx. Non-pathogenic *Neisseria* may have protective properties against some pathogens by producing secondary antimicrobial metabolites (Aho et al., 2020; Baerentsen et al., 2022). Some *Neisseria* species are also important for the development of a T-cell-independent polyclonal IgM response (Dorey et al., 2019). Many studies that compare the oropharyngeal microbiome of SARS-CoV-2-infected patients and healthy controls show a significant decrease in the representation of the genus *Neisseria* in the group of infected individuals (Li et al., 2022). The high abundance of nonpathogenic *Neisseria* in the oropharyngeal microbiome of SARS-CoV-2-infected patients is considered as a predictor of successful recovery (de Castilhos et al., 2022).

In our study, we observe overrepresentation of *Neisseria mucosa* and *Neisseria oralis* species in the group of severe patients. Our results are similar to the work where *Neisseria mucosa* was found to be associated with COVID-19 (Soffritti et al., 2021). Interestingly, we observed an opposite dynamic in the relative abundance of *Neisseria oralis* and *Neisseria mucosa* between the first and second time points in the mild group of patients, which may reflect a change of species at the same ecological niche. Moreover, Picrust2 analysis showed these species contribute significantly to the differentially represented metabolic pathways of the Type IV secretion system associated with the severe course of the disease. The type IV secretion system VirB/VirD4 is a major virulence determinant for subversion of human endothelial cell (HEC) function. (Schmid et al., 2004; Costa et al., 2021). According to the literature, the contribution of non-pathogenic *Neisseria* spp. to the prevalence of metabolic pathways associated with the type IV secretion system indicates that these genes, including virulence genes, are necessary for them to survive in the niche, but not for pathogenicity (Calder et al., 2020). Such genes are common to commensal and pathogenic *Neisseria* species and are necessary for the adhesion and invasion of bacterial cells to host cells and play an important role in the struggle for colonization within a given genus (Wörmann et al., 2016; Calder et al., 2020). In addition, members of the *Neisseria* genus are naturally competent, which increases the adaptability of the genus to changing environmental conditions such as inflammatory processes in the host organism and the use of antibiotics(Snyder et al., 2007; Goytia et al., 2021).

According to several studies, the members of the genus *Veillonella* strongly predominate in the oral microbiota of patients with Covid-19 compared to healthy controls (Iebba et al., 2021; Ma et al., 2021; Soffritti et al., 2021). Similar to *Capnocytophaga* spp., some *Veillonella* species associated with periodontitis have been overrepresented not only in the oral microbiome, but also in the BALF of the COVID-19 patients with pneumonia (Wu et al., 2020).

*Lautropia mirabilis* is normally isolated from human dental plaque and also from a supragingival or subgingival biofilm (Ikeda et al., 2020). To date, we have not found any information on the relationship of *Lautropia mirabilis* with COVID-19.

There are many studies worldwide describing the features of respiratory biotope in COVID-19 patients. Nevertheless, our study has several important strengths, such as the inclusion of patients from more than one geographic region, whose oropharyngeal swabs were collected during all seasons. In further comparison analysis we included only hospitalized patients whose swabs were taken at admission; therefore, we were more likely to capture the early upper respiratory tract microbiome associated with the development of severe COVID-19 rather than the one acquired after a hospital stay. We also had an opportunity to analyze samples collected from the same patients on the day of release. To our knowledge, this is the first study of the upper respiratory tract microbiome of COVID-19 patients from Russia.

However, we are aware of the objective limitations of our study leading to the sequencing batches (Wang et al., 2020; de Goffau et al., 2021). During this study we did everything to avoid it at sample processing level - all samples were processed the same way and sequenced simultaneously. To account for batch effects, we incorporated a categorical variable (batch) when calculating differential abundance of taxa and functions. Instead of using batch correction or adjustment methods, we chose this approach because our primary variable of interest (severity) was evenly distributed across batches, and we wanted to explicitly include this information in the model as recommended in the literature (Nygaard et al., 2016). Anyway, the samples were collected in different medical institutions, by different staff, using different consumables. Unfortunately, we were unable to control the sample acquisition procedure at this stage due to the height of the pandemic and the limitations involved. We suspect that this factor may have had the most significant impact, but it is not feasible to trace its effect.

Although experimental validation is needed, in our study we hypothesize that environmental and host-related factors could be affecting the respiratory microbiota prior to viral infection, potentially compromising the immune response of the host against disease. Also, the upper respiratory tract microbiome acts as a reservoir of opportunistic pathogens, which descend to the lower parts of the respiratory tract causing the elevated inflammation and lung damage. According to our data, we could suspect *Veillonella* acting this way. As far as nonpathogenic *Neisseria* are concerned, their over-representation in a group of severe COVID-19 patients can be considered as a predictor for further successful recovery in accordance with (de Castilhos et al., 2022). We can also assume that the microbiome of patients from the severe group recovers more slowly than from the mild one. It is evidenced by an increased relative abundance of opportunistic microorganisms, such as *Veilonella* sp., against an increased relative abundance of commensal flora, such as *Capnocytophaga sputigena* and *Haemophilus parahaemolyticus*, at the time of discharge from hospital.

It is worth mentioning that the comparative analysis of the relative abundances of certain bacteria in the oropharyngeal microbiome of mild and severe COVID-19 patients is rather ambiguous due to the complexity of the mechanisms leading to the observed trends. We believe that the findings of this work contribute to our understanding of the role of the upper respiratory tract microbiome in the development of COVID-19. Anyway, further studies that would shed light on the specific mechanisms through which the bacteria from respiratory microbiota are involved in host response against viral infections are required.

## Data availability statement

The datasets presented in this study can be found in online repositories. The names of the repository/repositories and accession number(s) can be found below: https://www.ncbi.nlm.nih.gov/, PRJNA751478.

## Ethics statement

The study was approved by the ethical committee of RCPCM. All patients gave written informed consent for sample collection and personal data processing. The patients/participants provided their written informed consent to participate in this study.

## Author contributions

JG, DF, AM, DKo, DKr, performed bioinformatics analyses. EI, AP, IG and VG conceived and supervised the study. ES, JG, EI wrote the manuscript. VB, KK, VV, MM and RG obtained samples and epidemiological data from patients and performed sample sequencing. All authors contributed to the article and approved the submitted version.
