# Supplementary figures and images for: Microbial communities of the upper respiratory tract in mild and severe COVID-19 patients: a possible link with the disease course
Source: Front Microbiomes. 2023 May 29;2:1067019. doi: 10.3389/frmbi.2023.1067019 (PMC12993638; doi:10.3389/frmbi.2023.1067019)

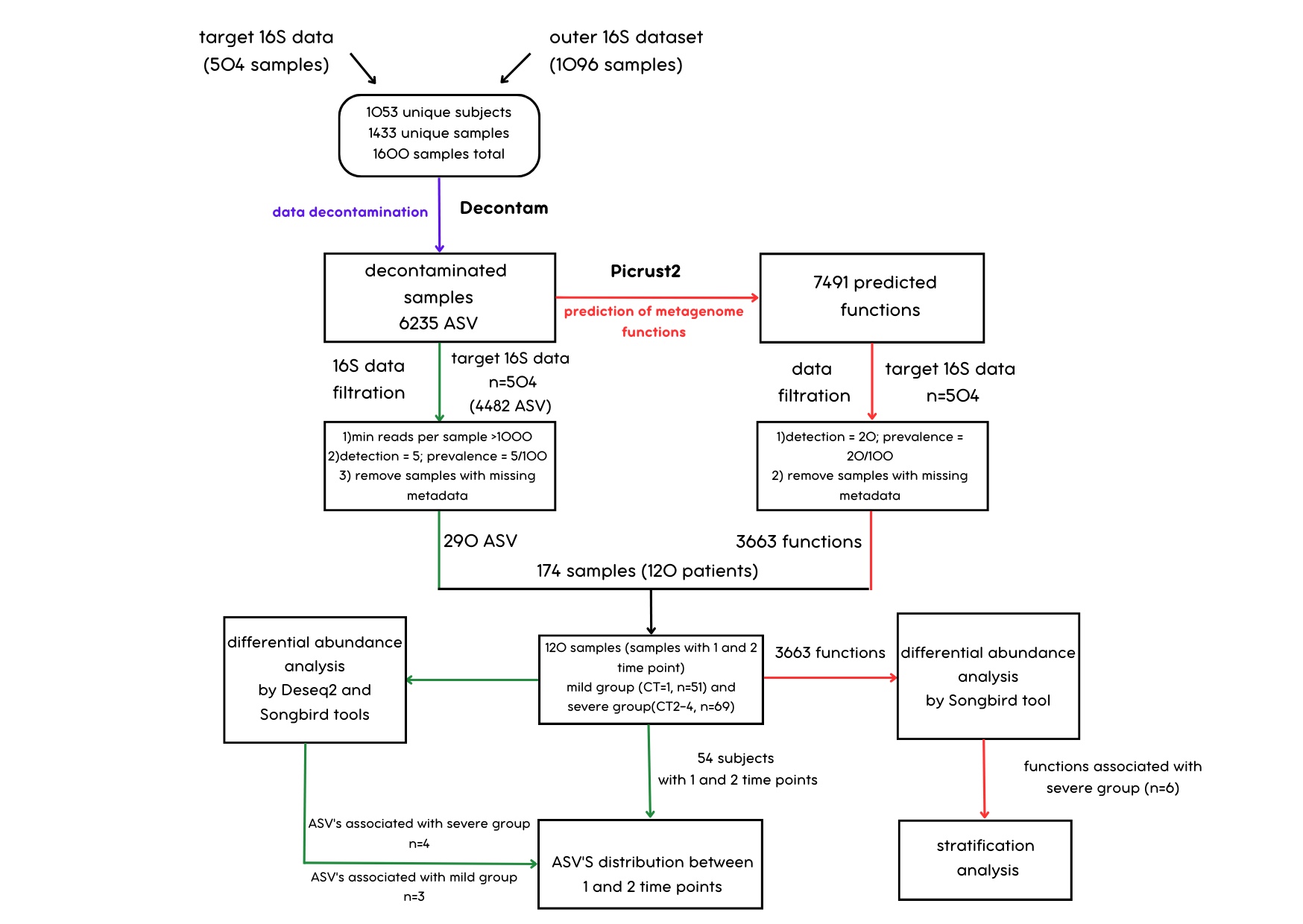

Supplement: Supplementary file 1 [file Image_1.jpeg]

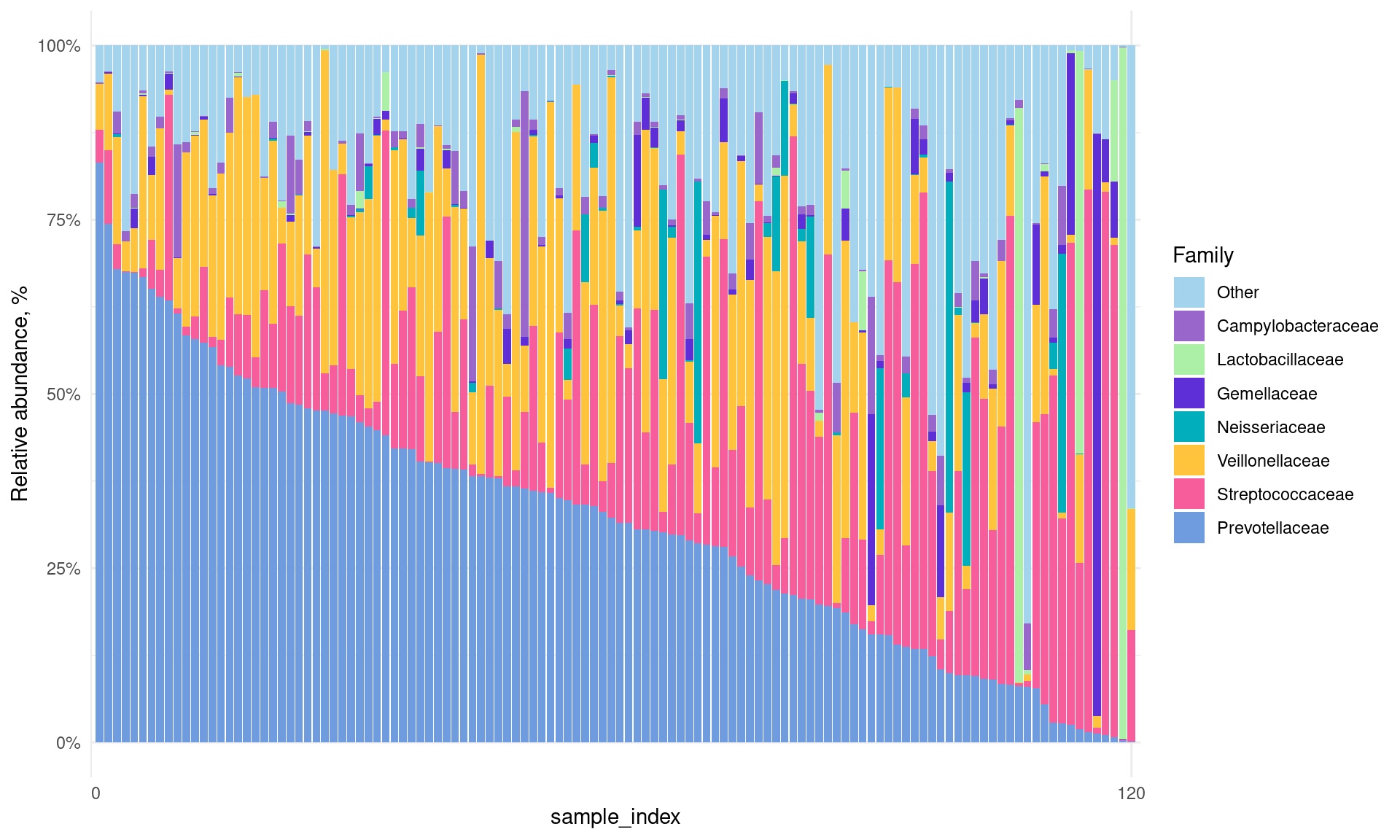

Supplement: Supplementary file 2 [file Image_2.jpeg]

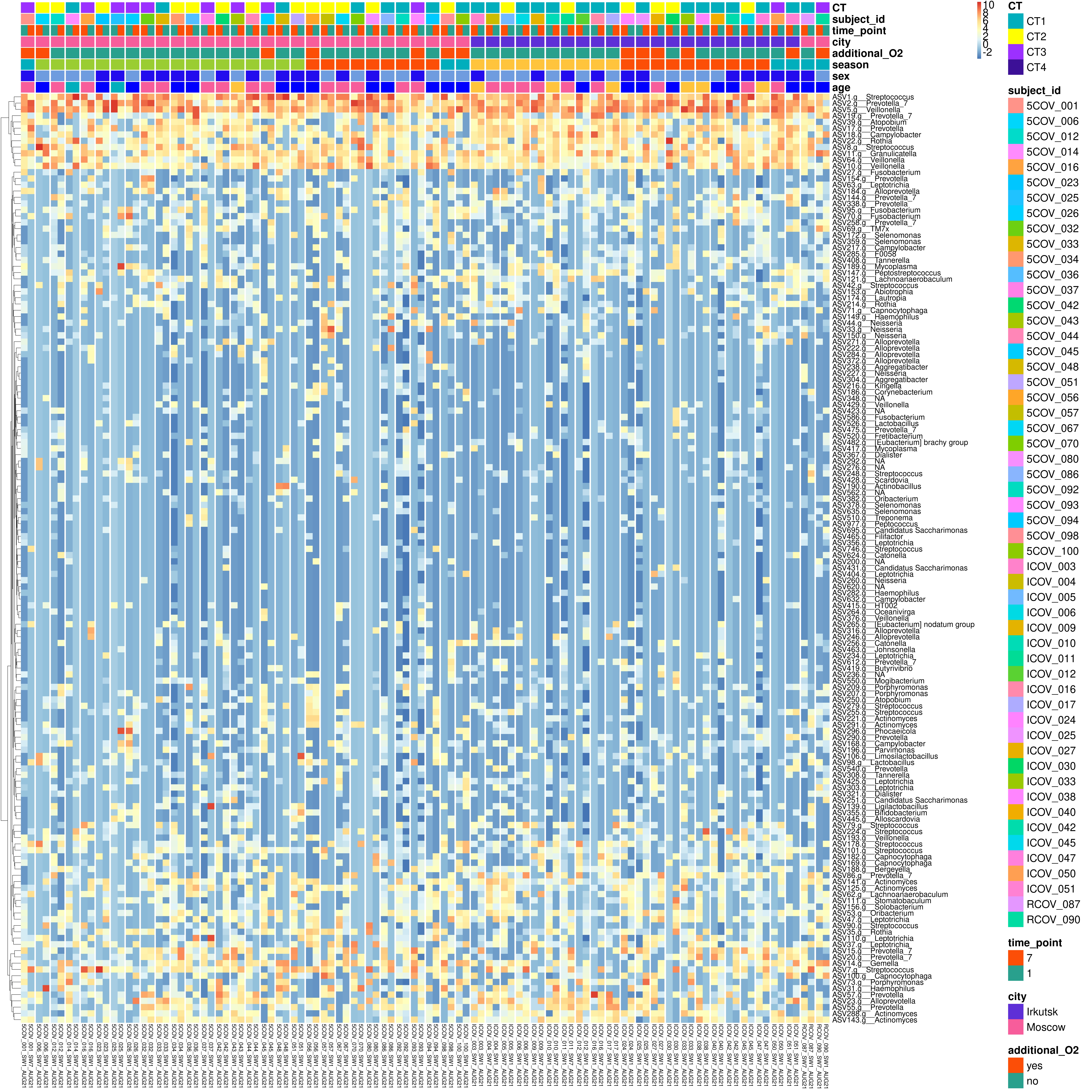

Supplement: Supplementary file 4 [file Image_4.jpeg]

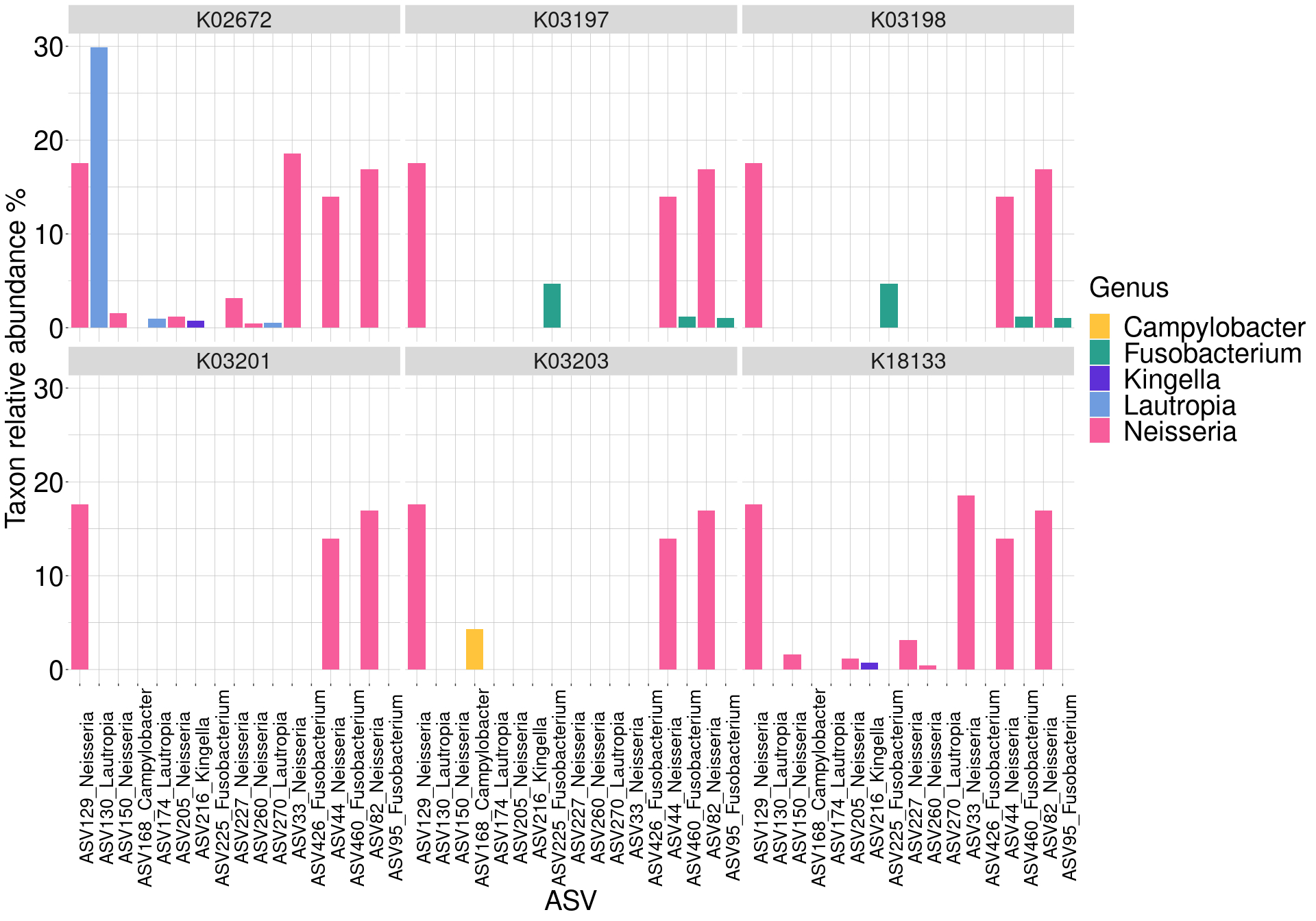

Supplement: Supplementary file 5 [file Image_5.jpeg]
