# Supplementary material for: Microbial communities of the upper respiratory tract in mild and severe COVID-19 patients: a possible link with the disease course
Source: Front Microbiomes. 2023 May 29;2:1067019. doi: 10.3389/frmbi.2023.1067019 (PMC12993638; doi:10.3389/frmbi.2023.1067019)
Supplement: Supplementary file 6 [file Table_1.docx]

Supplementary table1**.The results of aligning ASV sequences on nt database using BLASTN**

| **ASV** | **seq** | **per.ident,%** | **acc .len** | **genus** | **species** | **association** |
| --- | --- | --- | --- | --- | --- | --- |
| **ASV44** | **TGGGGAATTTTGGACAATGGGCGCAAGCCTGATCCAGCCATGCCGCGTGTCTGAAGAAGGCCTTCGGGTTGTAAAGGACTTTTGTCAGGGAAGAAAAGGCTGTTGCTAATACCGACAGCTGATGACGGTACCTGAAGAATAAGCACCGGCTAACTACGTGCCAGCAGCCGCGGTAATACGTAGGGTGCGAGCGTTAATCGGAATTACTGGGCGTAAAGCGAGCGCAGACGGTTACTTAAGCAGGATGTGAAATCCCCGGGCTCAACCTGGGAACTGCGTTCTGAACTGGGTGACTAGAGTGTGTCAGAGGGAGGTAGAATTCCACGTGTAGCAGTGAAATGCGTAGAGATGTGGAGGAATACCGATGGCGAAGGCAGCCTCCTGGGATAACACTGACGTTCATGCTCGAAAGCGTGGGTAGCAAACA** | **100** | **2224757** | **Neisseria** | **mucosa** | **severe** |
| **ASV150** | **TGGGGAATTTTGGACAATGGGGGCAACCCTGATCCAGCCATGCCGCGTGTCTGAAGAAGGCCTTCGGGTTGTAAAGGACTTTTGTCCGGGAAGAAAAGCGCGATGTTAATACCATTGCGTGCTGACGGTACCGGAAGAATAAGCACCGGCTAACTACGTGCCAGCAGCCGCGGTAATACGTAGGGTGCGAGCGTTAATCGGAATTACTGGGCGTAAAGCGGGCGCAGACGGTTACTTAAGCAGGATGTGAAATCCCCGGGCTCAACCTGGGAATTGCGTTCTGAACTGGGTGGCTAGAGTGTGTCAGAGGGGGGTAGAATTCCACGTGTAGCAGTGAAATGCGTAGAGATGTGGAGGAATACCGATGGCGAAGGCAGCCCCCTGGGATAGCACTGACGTTCATGCCCGAAAGCGTGGGTAGCAAACA** | **99.77** | **1472** | **Neisseria** | **oralis** | **severe** |
|  |  | **100** | **2502158** | **Neisseria** | **unidentified Neisseria sp** | **severe** |
| **ASV193** | **TGGGGAATCTTCCGCAATGGACGAAAGTCTGACGGAGCAACGCCGCGTGAGTGATGACGGCCTTCGGGTTGTAAAGCTCTGTTAATCGGGACGAAAGGCCTTCTTGCGAATAGTTAGAAGGATTGACGGTACCGGAATAGAAAGCCACGGCTAACTACGTGCCAGCAGCCGCGGTAATACGTAGGTGGCAAGCGTTGTCCGGAATTATTGGGCGTAAAGCGCGCGCAGGCGGATCAGTCAGTCTGTCTTAAAAGTTCGGGGCTTAACCCCGTGAGGGGATGGAAACTGCTGATCTAGAGTATCGGAGAGGAAAGTGGAATTCCTAGTGTAGCGGTGAAATGCGTAGATATTAGGAAGAACACCAGTGGCGAAGGCGACTTTCTGGACGAAAACTGACGCTGAGGCGCGAAAGCCAGGGGAGCGAACG** | **100** | **1485** | **Veillonella** | **uncultured bacterium** | **severe** |
| **ASV174** | **TGGGGAATTTTGGACAATGGGCGAAAGCCTGATCCAGCAATGCCGCGTGTGTGATGAAGGCCTTCGGGTTGTAAAGCACTTTTGGCGGGAACGAAAAGGACCGTGCCAATACCATGGTCTGATGACGGTACCCGCAGAATAAGCACCGGCTAACTACGTGCCAGCAGCCGCGGTAATACGTAGGGTGCAAGCGTTAATCGGAATTACTGGGCGTAAAGAGTGCGCAGGCGGTTTTGCAAGACCGATGTGAAATCCCCGGGCTTAACCTGGGAACTGCATTGGTGACTGCAAGGCTAGAGTGTGTCAGAGGGAGGTGGAATTCCGCATGTAGCAGTGAAATGCGTAGATATGCGGAGGAACACCGATGGCGAAGGCAGCCTCCTGGGATAACACTGACGCTCATGCACGAAAGCGTGGGGAGCAAACA** | **99.6** | **3172010** | **Lautropia** | **mirabilis** | **severe** |
|  |  | **100** | **1495** | **Lautropia** | **uncultured bacterium** |  |
| **ASV7** | **TAGGGAATCTTCGGCAATGGGGGCAACCCTGACCGAGCAACGCCGCGTGAGTGAAGAAGGTTTTCGGATCGTAAAGCTCTGTTGTAAGTCAAGAACGAGTGTGAGAGTGGAAAGTTCACACTGTGACGGTAGCTTACCAGAAAGGGACGGCTAACTACGTGCCAGCAGCCGCGGTAATACGTAGGTCCCGAGCGTTGTCCGGATTTATTGGGCGTAAAGCGAGCGCAGGCGGTTTGATAAGTCTGAAGTTAAAGGCTGTGGCTCAACCATAGTTCGCTTTGGAAACTGTCAAACTTGAGTGCAGAAGGGGAGAGTGGAATTCCATGTGTAGCGGTGAAATGCGTAGATATATGGAGGAACACCGGTGGCGAAAGCGGCTCTCTGGTCTGTAACTGACGCTGAGGCTCGAAAGCGTGGGGAGCGAACA** | **100** | **2096969** | **Streptococcus** | **salivarius** | **mild** |
| **ASV190** | **TGGGGAATATTGCACAATGGGGGGAACCCTGATGCAGCCATGCCGCGTGAATGAAGAAGGCCTTCGGGTTGTAAAGTTCTTTCGGTGACGAGGAAGGTTGATAGGTTAATAGCCTATCAAATTGACGTTAATCACAGAAGAAGCACCGGCTAACTCCGTGCCAGCAGCCGCGGTAATACGGGGGGTGCGAGCGTTAATCGGAATAACTGGGCGTAAAGGGCACGCAGGCGGTGACTTAAGTGAGATGTGAAAGCCCCGAGCTTAACTTGGGAATTGCATTTCATACTGGGTCGCTAGAGTACTTTAGGGAGGGGTAGAATTCCACGTGTAGCGGTGAAATGCGTAGAGATGTGGAGGAATACCGAAGGCGAAGGCAGCCCCTTGGGAATGTACTGACGCTCATGTGCGAAAGCGTGGGGAGCAAACA** | **100** | **2090133** | **Haemophilus** | **parahaemolyticus** | **mild** |
| **ASV71** | **TGAGGAATATTGGACAATGGTCGGAAGACTGATCCAGCCATGCCGCGTGCAGGATGAAGGTCCTATGGATTGTAAACTGCTTTTGTAAGGGAAGAATAAGGAGTACGTGTACTTTGATGACGGTACCTTACGAATAAGCATCGGCTAACTCCGTGCCAGCAGCCGCGGTAATACGGAGGATGCGAGCGTTATCCGGAATCATTGGGTTTAAAGGGTCCGTAGGCGGGCTGATAAGTCAGAGGTGAAAGCGCTTAGCTCAACTAAGCAACTGCCTTTGAAACTGTCAGTCTTGAATGATTGTGAAGTAGTTGGAATGTGTAGTGTAGCGGTGAAATGCTTAGATATTACACAGAACACCGATAGCGAAGGCATATTACTAACAATTTATTGACGCTGATGGACGAAAGCGTGGGGAGCGAACA** | **100** | **2933908** | **Capnocytophaga** | **sputigena** | **mild** |
